# Developing Pedestrians’ Red-light Violation Behavior Questionnaire (PRVBQ); Assessment of Content Validity and Reliability

**DOI:** 10.30476/BEAT.2020.46449

**Published:** 2020-04

**Authors:** Mahdi Moshki, Abdoljavad Khajavi, Homayoun Sadeghi-Bazargani, Shahram Vahedi, Saeid Pour-Doulati

**Affiliations:** 1 *Department of Health Education and Health Promotion, School of Health Sciences; Social Development and Health Promotion Research Center, Gonabad University of Medical Sciences, Gonabad, Iran*; 2 *Community Medicine Department, School of Medicine, Gonabad University of Medical Sciences, Gonabad, Iran*; 3 *Road Traffic Injury Research Center, Tabriz University ofMedical Sciences, Tabriz, Iran*; 4 *Department of Education and Psychology, University of Tabriz, Tabriz, Iran*; 5 *Social Development and Health Promotion Research Center, Gonabad University of Medical Sciences, Gonabad, Iran*

**Keywords:** Pedestrian red-light violation behavior questionnaire, Validity, Reliability

## Abstract

**Objective:**

To develop a self-completion pedestrians’ red-light violation behavior questionnaire (PRVBQ) based on the theory of planned behavior (TPB) and assess the content validity and reliability.

**Methods:**

This study was conducted in three phases of (i) PRVBQ development study; (ii) Content validity study including face validity; and (iii) Reliability assessment. The directed content analysis method was used for the analysis of the qualitative interviews. The item impact score was used for face validity. Content validity index (CVI) in the item level and average scale level, and content validity ratio (CVR) were determined. Intra-class Correlation Coefficient (ICC), and Cronbach’s alpha was assessed for test-retest reliability and internal consistency respectively.

**Results:**

Draft questionnaire including 86 items was constructed. Sixteen items were eliminated due to low face and content validity, remaining 70 items in total. The PRVBQ was rated as having good content validity (individual items CVI ranged from .80 to 1, and overall PRVBQ CVI-Average=0.95, *p*=0.05). The direct measures (reflective indicators) showed excellent internal consistency with Cronbach’s alpha=0.9. All items showed excellent agreement.

**Conclusion:**

This study using a comprehensive process of development and assessment of content validity and reliability developed a content valid and reliable questionnaire predicting pedestrians’ red light violation behavior.

## Introduction

The increasing growth of motor vehicles and insufficient attention to pedestrian safety have put them at risk of injury, disability, and death. Pedestrians account for 23 percent of road traffic deaths worldwide. Pedestrians, owing to having no shield at all to protect them in case of a collision and having very low mass in comparison with motorized vehicles are more vulnerable than other road users [1]. Prevention of pedestrian injuries is an important policy of the health care systems. Evidence suggests that pedestrian injuries are both predictable and preventable [2].

Developing effective prevention strategies require gaining deep understanding of traffic accident causes. Human component is the main cause of traffic accidents in contrast with environment and vehicle as the other two components of traffic accidents. Prohibited road-crossings at signalized intersections, where automatic traffic signals indicate to pedestrians; when they should cross, and was known as the most common violations of traffic rules. Pedestrians in developing countries are more likely to commit a traffic violation and display more risky behaviors [3].

Therefore, investigating the underlying factors of pedestrians’ risky road-crossing behaviors is essential to develop an evidence-based and effective intervention. The theory of planned behavior (TPB; [4-7] ) is a widely used socio-psychological model helping researches and safety intervention planners to understand pedestrians’ unsafe road crossing behavior [3, 8-14]. Based on the TPB, behavioral intention and perceived behavioral control (PBC) are proximal predictors of actual behavior. In this model, three latent variables including attitude (ATT) toward the behavior, subjective norms (SN), and PBC predict intention to perform a behavior. "Intention" is the antecedent variable of behavior. The latent variables are needed to be measured indirectly by questionnaire responses [15]. 

TPB based questionnaires may include direct measures (i.e., reflective indicators), indirect measures (i.e., formative indicators), or both measures for each latent variable of ATT, SN, and PBC. Indirect measures are constructed based on the expectancy value theory [16]. The direct measures, e.g. ask respondents about the opinion of important people in general and indirect measures, e.g. ask about the strength of normative beliefs with respect to each reference group and motivation to comply with them. Direct measures used for the prediction and indirect measures used for determining the underlying beliefs of specific behaviors, but neither approach is perfect. Therefore, it is recommended that each TPB questionnaire uses both direct and indirect measures [15]. 

In addition to the main constructs of the TPB, past behavior (PB) is a variable that play a significant role in explaining pedestrian’s unsafe road crossing behavioral intention. The habitual behavior is a mental concept that can be automatically triggered by the environment. Repeating a previous behavior strengthens the habit. Therefore, past behavior, along with the ATT, SN, and BPC can contribute to behavioral intention, which in turn determines the future behavior, past behavior, explaining 14 percent of the variance in a pedestrians’ intention to jaywalk, was introduced as the strongest predictor of the pedestrians' unsafe road crossing behavioral intention in China [12].

Considering the high rate of pedestrian injury and mortality and their unsafe road crossing behavior as a major risk factor, investigating the pedestrians' risky crossing behavior for developing effective preventive interventions is highly felt. Understanding the reasons for pedestrians’ unsafe road crossing behavior needs to develop valid and reliable measures [2]. Development of a valid and reliable measurement instrument is a very critical point, particularly in social-psychological and health-related behavior research. Validity ensures that the measurement instrument is measuring what it anticipates to determine and is reflecting the intended theoretical concept [17].

Evaluating content validity (including face validity) of a measurement instrument is the most important and a critical early step in the construct validity of an instrument [18]. Content validity refers to the degree to which items of a measurement instrument adequately represent the content domain. If an instrument lacks content validity, it is impossible to establish reliability for it [19]. Most TPB based questionnaires used for predicting pedestrians’ unsafe road crossing behavior have not presented sufficient evidence of validity and reliability. Therefore, the purpose of the present study was to develop a self-completion pedestrians’ red-light violation behavior questionnaire (PRVBQ) based on the TPB and the extended variable (PB), to use it for the predictive application and to assess the content validity and reliability of scores in the sample of adult pedestrians of Tabriz city, Iran (in Persian).

## Materials and Methods

This study was part of a larger study approved by the Local Ethics Committee and research council of Gonabad University of Medical Sciences. Gonabad, Iran (approval code: IR.GMU.REC.195.19). The present study was a sequential exploratory mixed method (qualitative and quantitative) that took place in three phases between Jun 2016 and November 2017 and was conducted in Tabriz, Iran.

***Construct***

**Fig. 1:**
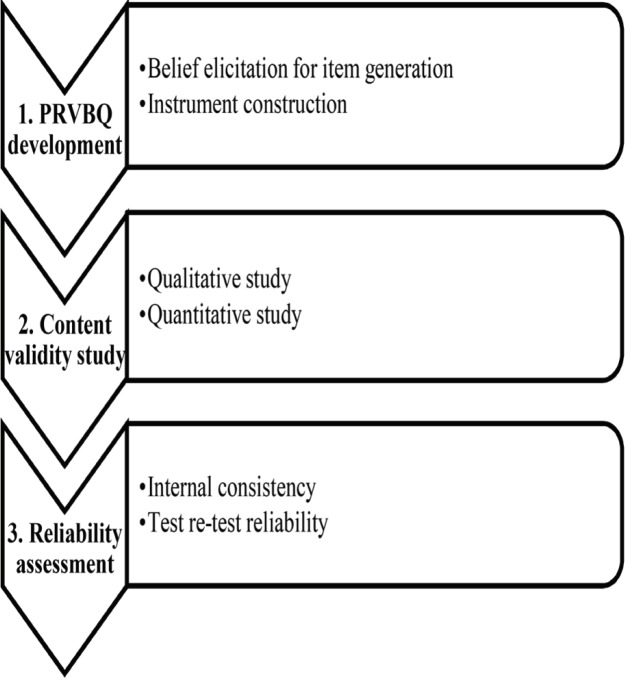
Three phases of content validity and reliability study

The construct of the study is PRVB, which has the potential of crash, leading to injury and death. This study was conducted in 3 phases (Figure 1) including (i) PRVBQ development study consisted of belief elicitation for item generation and instrument construction; (ii) Content validity study including face validity; and (iii) Reliability assessment including internal consistency and test re-test reliability.

***Phase 1: PRVBQ development study including belief elicitation for item generation and instrument construction***

**Fig. 2:**
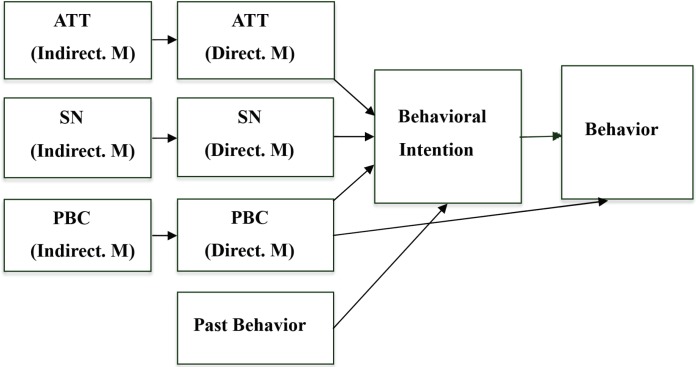
Conceptual model used for PRVBQ development

This qualitative study provided the relevancy and comprehensives of the items being used in the questionnaire. Thirty pedestrians using semi-structured open-ended questions were interviewed to elicit salient outcomes, social referents, and circumstances regarding pedestrians’ traffic light violation. Interviews were continued until saturation was reached. Directed content analysis was used by two independent skilled coders to analyze the transcribed interviews and capture salient beliefs. Then, the beliefs obtained from the directed content analysis were subjected to the frequency analysis. The rule of selecting the beliefs expressed by at least 10 percent of the respondents (one of the three rules suggested by Aizen and Fishbein) was applied to include the most frequent beliefs in the modal set (Detailed information has been provided elsewhere [20]). Based on the information obtained from this study and based on the manuals for constructing a TPB questionnaire provided by Aizen and Fishbein [16, 21], and Francis *et al*.’s study [15], the first draft of PRVBQ was constructed. The main constructs of the TPB consisted of ATT, SN, PBC, and behavioral intention (BI), plus extended construct of PB, that were used for items formulation. Since neither approach of direct and indirect measures was perfect, we used both in developing PRVBQ for the prediction of PRVB (Figure 2).

***Phase 2: Content validity study including face validity***

***1- Qualitative evaluating face and content validity of the PRVBQ (Cognitive interview study)***

This stage provided information on the comprehensiveness and especially the comprehensibility and relevancy of the items. First, four of colleagues were asked to fill in the questionnaire. We provided them a cover letter consisted of a brief description of research, study title and its objectives, conceptual model, its dimensions, and instructions on how to fill in the questionnaire. These provisions have also been provided in other stages of the content validity and reliability study, where we have provided the questionnaire to pedestrians or experts. Then, the entire questionnaire was presented requesting them to review the draft PRVBQ and comment on relevancy, comprehensiveness, and appropriateness of items to the target population. After adaptation based on their feedbacks, eight people who were similar to the target population drew up for the cognitive interview. The target population was urban pedestrians living in the Tabriz city, aged≥18 years, lacking physical and mental disabilities. We used concurrent approach for cognitive interview. A skilled interviewer based on the predefined interview guide asked the interviewees to complete the questionnaire and asked them about their understanding of the PRVBQ instructions, items and response options to determine manifest complications over items for testing the comprehensibility, relevancy, acceptability and feasibility of the measurement instrument. The technics of think aloud (respondents verbalized their thoughts while reading each question and chose the answer) and paraphrasing (respondents were asked to rephrase an item in his/her own words) were employed for understanding. if the item was misunderstood and could be rephrased. Participants provided many recommendations to further improvement of the face and content validity of the PRVBQ.

In order to ensure rigor of the analyses and prevent bias, two independent researchers were involved in the analyses. All interviews were audio recorded and transcribed verbatim. We made necessary modifications on the interview guide, questionnaire items, response options and recall period based on the respondents’ comments from the first round of the cognitive interviews. Following the first round of cognitive interview, an expert’s panel provided a cover letter and the entire questionnaire to review the PRVBQ and comment on relevancy, comprehensiveness, and appropriateness to the target population. Necessary modifications were made on the PRVBQ based on the expert comments. The second round of cognitive interview with eight pedestrians was conducted to confirm the face and content validity. In this round, based on the respondents’ comments, minor revisions were made. Then the revised questionnaire was subjected to the quantitative face and content validity. 

*2- Quantitative evaluating of face and content validity of the PRVBQ*

*2-1- Face validity*

A group of pedestrians (N=10) after providing them a cover letter were requested to rate the importance of each item on a 5-point Likert scale. The item impact score (frequency × importance) was calculated. The impact score ≥1.5 was considered acceptable [22].

*2-2- Content validity*

Quantitative content validity was calculated by measuring content validity index (CVI) based on Waltz and Bausell approach [23], and the content validity ratio (CVR). CVI, the most widely reported approach for content validity, was first calculated [24]. An expert panel (N=10) in the field of road safety, health promotion, and psychometric was requested to score the relevance, and comprehensibility (in terms of clearness and simplicity) of each item using a 4-point ordinal rating scale e.g. for relevancy, (1: Not relevant, 2: Somehow relevant, 3: Quite relevant, and 4: Highly relevant). Item level content validity index (I‑CVI) was calculated by dividing the number of experts who gave each item scores 3 or 4 by the total number of experts participating in the panel. Having three different I-CVI for each item in terms of relevancy, clarity and simplicity; we computed the average I-CVI for each item by adding three I-CVIs, and divided by three. According to Lynn’s criteria, if the number of experts is between 6 and 10, I-CVI equal to or higher than 0.78 is considered to be excellent [25]. Items with an I-CVI between 0.70 to 0.78 were revised and items with a I-CVI lower than 0.70 were deleted [18]. Scale level content validity index (S-CVI/Ave) was calculated by adding together the items with I-CVI above 0.78 and divided by the total number of items. S‑CVI/Ave≥0.9 was considered acceptable according to the recommendation of Polit *et al.* [18]. As substantial item improvement not necessitated, and all aspects of the construct adequately covered by the initial pool of items, the second round of CVI study was not conducted. Remaining items were subjected to the CVR study. Then, CVR was calculated for each item. CVR specifies whether an item is necessary to be included in the questionnaire or not. An expert panel (N=8) was requested to specify whether an item is necessary for operating a construct in a set of items or not by specifying each item as “not necessary, useful but not essential, or essential”. CVR was calculated by the following formula: CVR=(Ne-N/2)/(N/2), in which the Ne is the number of panelists indicating "essential" and N is the total number of panelists. Lawshe table was used for determining the numeric value of the CVR. Having eight experts, a minimum value of 0.75 was considered an acceptable level of significance.

***Phase 3- Reliability***

**Internal consistency and temporal reliability assessed for the reliability of the instrument.**

***1-Temporal reliability***

Test re-test reliability was used for assessing temporal reliability. Fifty pedestrians over a two-week interval answered the questionnaire. The correlation between the individual questions, demonstrated the stability of the instrument. For assessing the temporal stability, Intra-class Correlation Coefficient (ICC) was calculated for each item. According to Cicchetti [26], Cicchetti and Sparrow [27], and Fleiss [28], ICC values were interpreted for the reliability as follows: values≥0.74 were considered excellent, values from 0.60 to 0.74 were depicted good, values from 0.40 to 0.59 were regarded fair, and values≤0.40 were illustrated poor.

**Fig. 3:**
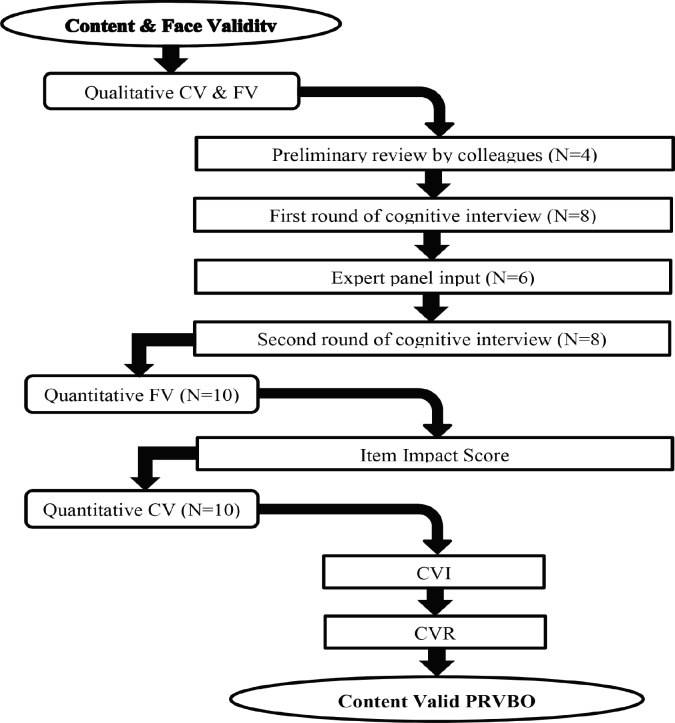
Content validity study flowchart

***2- Equivalence reliability***

As noted earlier, we included direct and indirect measures (i.e., reflective and formative indicators, respectively) in the PRVBQ. Since the behavioral, normative, and control belief composites were formative indicators of ATT, SN, and PBC, respectively, there was no requirement of internal consistency for them. Therefore, we calculated internal consistency only for direct measures. Cronbach's alpha coefficient, the most widely used statistic to assess the internal consistency of a scale, indicated how well the items on a tool jointly measure the same construct [29, 30]. To test internal consistency, 50 pedestrians completed a copy of the questionnaire. We calculated Cronbach’s alpha coefficient to assess the internal consistency of the entire questionnaire and for each subscale. Values equal or above 0.7 were considered as acceptable level [26, 31]. Cronbach’s alpha and ICC were calculated using the Statistical Package for Social Sciences (SPSS, version 24.0, Chicago, IL, USA) at a significance level of 0.05.

## Results

***Phase 1- PRVBQ development study consists of belief elicitation for item generation and draft instrument construction***

The demographic characteristics of the belief elicitation participants can be found in Table 1.

**Table 1:** The demographic characteristics of the belief elicitation participants

Age (N=30)		Sex (N=30)		Education (N=30)		Occupation (N=30)		Marriage (N=30)		Crossing (N=30)	
**Mean**	4.93	Male	15	Under diploma	1	Public employee	14	Married	21	Cross	15
**Median**	39.00	Female	15	Diploma	6	Private employee	2	Single	9	Not cross	15
**SD**	13.06			Associate degree	1	Self-employee	6				
**Min**	21			Bachelor degree	18	Retired	4				
**Max**	75			Master degree	3	Student	2				
				Doctorate	1	Housewife	2				

***Belief elicitation for item generation***

Four to twenty sub-categories were generated through directed content analysis for each of ten predefined categories of the TPB. Consistent with the TPB questionnaire, these categories were advantages, disadvantages, positive feelings, negative feelings, approving referents, disapproving referents, behaving referents, not-behaving referents, facilitators, and barriers. We added the elicited advantage beliefs and disadvantage beliefs together to create a set of instrumental beliefs. Similarly, to create a set of experiential beliefs we combined the beliefs of like and dislike together. 

Finally, all instrumental beliefs and experiential beliefs were put together to yield a set of attitudinal beliefs. Normative and control beliefs have been made like the same process of attitudinal beliefs. After producing attitudinal, normative and control beliefs, using the Aizen and Fishbein’s rule of “belief mentioned by at least 10% of participants”, the most frequently mentioned beliefs were identified to be used for crafting the primary draft of PRVBQ based on the TPB constructs and additional construct of “past behavior” comprised of 86 items in total.

The most important outcomes of the PRVB were getting injured, time saving, breaking the law, disturbing city system, lowering the level of culture, violating citizenship rights, and financial damage. The most important social referents were family members, friends, educated people, colloquies, relatives, and youth. The most important circumstances regarding PRVB were “Being in a hurry” “No police presence” “Fear of accident” “Complying with the law” “Not crossing other pedestrians” and “Physical ability”.

***Phase 2- Content validity study including face validity of the instrument***

***Qualitative evaluating face and content validity of the PRVBQ (Cognitive interview study)***

First Cognitive interview study

Cognitive debriefing of the interviews indicated that the PRVBQ was relevant to the PRVB, but many items were needed to be revised to improve the comprehensiveness of items. Response options also recognized problematic and needed to be revised to produce valid responses by the pedestrians. To further improve the face and content validity of the PRVBQ, pedestrians provided many constructive suggestions. The revisions were made to fulfil these recommendations.

Expert panel input

An expert panel reviewed the revised PRVBQ and made some critical comments on relevancy, comprehensiveness, and appropriateness to the target population. Based on their inputs necessary modifications made on the PRVBQ to increase the relevancy and comprehensiveness of the instrument. 

Second cognitive interview study

Overall, the revised PRVBQ, like the first round of cognitive debriefing interviews, was relevant to the participant pedestrians. Although this version was more comprehensive and acceptable than the initial draft version, but a few minor problems were detected in this version, likely due to the complexity of employed conceptual framework. So we made necessary modifications to the instrument to increase the comprehensibility of the instructions, items and response options of the instrument. The revised PRVBQ with 86 items was subjected to the quantitative content validity including face validity. 

*Quantitative evaluating of face and content validity of the PRVBQ*

**Face validity**

*All 86 items of the questionnaire having impact score higher than 1.5 were retained and subjected to the next step of content validity (* Table 2 *).*

**Table 2:** Items of PRVBQ Scales before and after face and content validity

Item deletion	(N*)			Domain	Sub-scale		Scale	Measures
After CVR	**After CVI**	**After FV**	**Initial Items**					
**5**	5	7	7		Behavioral belief Strength	Attitude		Direct Measures
**5**	5	7	7	Cognitive	Outcome evaluations			
**5**	5	7	7	Affect				
**5**	7	7	7		Injunctive belief Strength	Injunctive Norm		
**5**	7	7	7		Motivation to comply			
**5**	7	7	7		Descriptive belief Strength	Descriptive norm		
**5**	7	7	7		Identification with Referent			
**7**	7	8	8		Control belief strength	Perceived Behavioral Control		
**7**	7	8	8		Power of control Factors			
**4**	4	4	4		Direct attitude	Attitude		Indirect Measures
**4**	4	4	4		Direct perceived norm	Subjective norm		
**3**	3	3	3	Self-efficacy	Direct perceived Control	Perceived Behavioral Control		
**3**	3	3	3	Autonomy				
**4**	4	4	4		Behavioral intention	Behavioral Intention		
**3**	3	3	3		Past behavior	Past behavior		Extended Measure
**70**	78	86	86	Total items				

***Content Validity Index (*** *CVI)*

Based on the results of the I-CVI for all 86 items of the questionnaire, eight items (i.e., the items 3, 6, 10, 13, 17, 21, 57, and 65), having I-CVI of less than 0.78 were excluded after careful consideration. The I-CVI of the 78 remaining items was between 0.8 and 1 with S-CVI/Ave equal to 0.95.

***Content Validity Ratio (*** *CVR)*

Eight items (i.e., the items 27, 28, 34, 35, 41, 42, 48, 49) having CVR less than 0.75 were excluded from the questionnaire, remaining 70 items to the reliability study (Table 2).

***Phase 3: Reliability assessment including internal consistency and test re-test reliability***

The Cronbach’s alpha reliability coefficient for the collective 22-item direct measures was 0.90, indicating excellent internal consistency reliability, ranged from 0.83 to 0.97. All items showed excellent agreement, ICC=0.88 (95% CI [0.80, 0.93]).

## Discussion

The present study demonstrated development of a new questionnaire to understand pedestrians’ red-light violation behavior and assessment of content validity and reliability of this instrument. Developing a measurement instrument with acceptable content validity is an iterative and lengthy process. This process started with instrument development including belief elicitation for item generation and instrument construction, followed by content validity study including face validity, and continued with reliability study.

Based on the qualitative belief elicitation study, in total, 22 beliefs concerning salient outcomes, social referents, and circumstances regarding pedestrians’ red light violation has been recognized. Using these beliefs and based on the TPB constructs and additional construct of PB, we formulated the initial form of questionnaire items comprised of 86 items. Previous nine studies of TPB based questionnaire studying pedestrian behavior, except one [9] have not reported conducting a belief elicitation study for item generation [3, 8, 10-14, 32].

PRVBQ 86 items was first subjected to the two-round qualitative cognitive interview with pedestrians and an expert panel in between. Based on the results of the two rounds interview and expert panel comments, most items and response options were subjected to thorough revision. Previous nine studies of TPB based questionnaire studying pedestrian behavior [3, 8-14, 32], except Barerro *et al.*, [8] have not reported conducting a cognitive interview for testing face and content validity.

Due to scrutinized item generation process and thorough revision of almost all 86 items after cognitive debriefing interview, none of the items was deleted due to item impact score lower than 1.5. Nine items of the questionnaire with I-CVI<0.78, and eight items with CVR<0.75 were excluded from the questionnaire. I-CVI for each item was assessed in terms of relevancy, clarity, and simplicity. Although the results of relevancy were similar to the clarity and simplicity, the results of the last two were quite similar. 

This is why many researchers only use relevancy to calculate CVI, not using clarity and simplicity. On the other hand, it can be said that these two criteria are carefully examined in the cognitive debriefing interviews and the necessary modifications have been already made. Cronbach’s alpha showed that 69 items PRVBQ had excellent internal consistency reliability. By conducting test re-test reliability, all items showed excellent temporal reliability. An excellent temporal reliability was obtained for the individual items of the questionnaire (ICC=0.88). 

We have also calculated ICC for composite beliefs and came up with ICC=0.79 (95% CI [0.66, 0.88]) indicating excellent composite beliefs agreement (Table 3). Zhou *et al*. have used only indirect measures of TPB and extended construct of perceived risk, and conformity tendency [12]. They reported internal consistency for behavioral intention and each sub-scale of indirect measures by Cronbach’s alpha ranging from 0.68 to 0.85. Barrero *et al*. reported Cronbach’s alpha above 0.7 for sub-constructs but, it was not high for the whole construct [8]. 

Hashemiparast *et al*. reported Cronbach’s alpha ranged from 0.67 to 0.88 and ICC ranged from 0.64 to 0.96 for the Pedestrian Road Crossing Behavior (PROB) questionnaire [32]. Xu *et al.* reported Cronbach’s alpha ranged from 0.77 to 0.92 [3]. This study developed a measurement instrument based on the belief elicitation for item generation and assessed content validity and reliability of the PRVBQ, but construct validity including structural validity and hypothesis testing using larger sample size was needed to be studied in the next step. This study used a comprehensive process developed a content valid and reliable questionnaire for predicting pedestrians’ red light violation behavior. Besides, this questionnaire could be used for determining the underlying factors of such risky behavior.

**Table 3:** Intra-class correlation coefficient (ICC) of the composite beliefs

P value	ICC	α	Item
**0.001**	0.675 (0.490-0.802)	0.806	IAT^a^1
**0.001**	0.774 (0.634-0.865)	0.873	IAT2
**0.001**	0.789 (0.656-0.874)	0.882	IAT3
**0.001**	0.692 (0.514-0.813)	0.818	IAT4
**0.001**	0.724 (0.560-0.834)	0.840	IAT5
**0.001**	0.725 (0.562-0.834)	0.841	EAT^b^1
**0.001**	0.793 (0.661-0.877)	0.884	EAT2
**0.001**	0.747 (0.593-0.848)	0.855	EAT3
**0.001**	0.691 (0.512-0.812)	0.817	EAT4
**0.001**	0.761 (0.641-0.857)	0.864	EAT5
**0.001**	0.640 (0.442-0.778)	0.780	INO^c^1
**0.001**	0.934 (0.886-0.962)	0.966	INO2
**0.001**	0.716 (0.548-0.828)	0.834	INO3
**0.001**	0.716 (0.549-0.828)	0.835	INO4
**0.001**	0.655 (0.462-0.788)	0.791	INO5
**0.001**	0.650 (0.456-0.785)	0.788	DNO^d^1
**0.001**	0.674 (0.489-0.801)	0.805	DNO2
**0.001**	0.788 (0.655-0.874)	0.882	DNO3
**0.001**	0.748 (0.595-0.849)	0.856	DNO4
**0.001**	0.708 (0.537-0.823)	0.829	DNO5
**0.001**	0.779 (0.641-0.868)	0.876	PBC^e^1
**0.001**	0.792 (0.661-0.871)	0.884	PBC2
**0.001**	0.633 (0.432-0.774)	0.775	PBC3
**0.001**	0.795 (0.664-0.878)	0.886	PBC4
**0.001**	0.837 (0.730-0.904)	0.911	PBC5
**0.001**	0.814 (0.693-0.890)	0.897	PBC6
**0.001**	0.778 (0.640-0.868)	0.875	PBC7

^a^Instrumental Attitude; ^b^Experiential Attitude; ^c^Injunctive Norms; ^d^Descriptive Norms; ^e^Perceived Behavioral Control.
